# Chloroquine and Sulfadoxine Derivatives Inhibit ZIKV Replication in Cervical Cells

**DOI:** 10.3390/v13010036

**Published:** 2020-12-29

**Authors:** Audrien Alves Andrade de Souza, Lauana Ribas Torres, Lyana Rodrigues Pinto Lima Capobianco, Vanessa Salete de Paula, Cynthia Machado Cascabulho, Kelly Salomão, Maria da Gloria Bonecini-Almeida, Maria de Lourdes Garcia Ferreira, Nubia Boechat, Luiz Carlos da Silva Pinheiro, Elen Mello de Souza

**Affiliations:** 1 Laboratório de Virologia Molecular, Instituto Oswaldo Cruz/FIOCRUZ, Rio de Janeiro, RJ 21040-360, Brazil; audrien.andrade@ioc.fiocruz.br (A.A.A.d.S.); lauana.torres@ioc.fiocruz.br (L.R.T.); lyana.rpl@gmail.com (L.R.P.L.C.); vdepaula@ioc.fiocruz.br (V.S.d.P.); 2 Laboratório de Inovações em Terapias, Ensino e Bioprodutos, Instituto Oswaldo Cruz/FIOCRUZ, Rio de Janeiro, RJ 21040-360, Brazil; cynthiac@ioc.fiocruz.br; 3 Laboratório de Biologia Celular, Instituto Oswaldo Cruz/FIOCRUZ, Rio de Janeiro, RJ 21040-360, Brazil; ks@ioc.fiocruz.br; 4 Laboratório de Imunologia e Imunogenética em Doenças Infecciosas, Instituto Nacional de Infectologia Evandro Chagas/FIOCRUZ, Rio de Janeiro, RJ 21040-360 Brazil; gloria.bonecini@ini.fiocruz.br; 5 Laboratório de Síntese de Fármacos, Instituto de Tecnologia em Fármacos, Farmanguinhos—FIOCRUZ, Rio de Janeiro, RJ 21040-360, Brazil; maluferreir@gmail.com (M.d.L.G.F.); nboechat@gmail.com (N.B.); pinheirolcs@gmail.com (L.C.d.S.P.)

**Keywords:** Zika virus, chloroquine, sulfadoxine, hybrid compounds, antiviral effect, human cervical cells

## Abstract

Despite the severe morbidity caused by Zika fever, its specific treatment is still a challenge for public health. Several research groups have investigated the drug repurposing of chloroquine. However, the highly toxic side effect induced by chloroquine paves the way for the improvement of this drug for use in Zika fever clinics. Our aim is to evaluate the anti-Zika virus (ZIKV) effect of hybrid compounds derived from chloroquine and sulfadoxine antimalarial drugs. The antiviral activity of hybrid compounds (C-Sd1 to C-Sd7) was assessed in an in-vitro model of human cervical and Vero cell lines infected with a Brazilian (BR) ZIKV strain. First, we evaluated the cytotoxic effect on cultures treated with up to 200 µM of C-Sds and observed CC_50_ values that ranged from 112.0 ± 1.8 to >200 µM in cervical cells and 43.2 ± 0.4 to 143.0 ± 1.3 µM in Vero cells. Then, the cultures were ZIKV-infected and treated with up to 25 µM of C-Sds for 48 h. The treatment of cervical cells with C-Sds at 12 µM induced a reduction of 79.8% ± 4.2% to 90.7% ± 1.5% of ZIKV–envelope glycoprotein expression in infected cells as compared to 36.8% ± 2.9% of infection in vehicle control. The viral load was also investigated and revealed a reduction of 2- to 3-logs of ZIKV genome copies/mL in culture supernatants compared to 6.7 ± 0.7 × 10^8^ copies/mL in vehicle control. The dose–response curve by plaque-forming reduction (PFR) in cervical cells revealed a potent dose-dependent activity of C-Sds in inhibiting ZIKV replication, with PFR above 50% and 90% at 6 and 12 µM, respectively, while 25 µM inhibited 100% of viral progeny. The treatment of Vero cells at 12 µM led to 100% PFR, confirming the C-Sds activity in another cell type. Regarding effective concentration in cervical cells, the EC_50_ values ranged from 3.2 ± 0.1 to 5.0 ± 0.2 µM, and the EC_90_ values ranged from 7.2 ± 0.1 to 11.6 ± 0.1 µM, with selectivity index above 40 for most C-Sds, showing a good therapeutic window. Here, our aim is to investigate the anti-ZIKV activity of new hybrid compounds that show highly potent efficacy as inhibitors of ZIKV in-vitro infection. However, further studies will be needed to investigate whether these new chemical structures can lead to the improvement of chloroquine antiviral activity.

## 1. Introduction

The Zika virus (ZIKV) circulates in the Americas, Africa, Southeast Asia, and the Pacific Islands. In the American continent alone, there have been 223,477 confirmed autochthonous cases of Zika disease [1,2]. Its infection usually leads to a mild and self-limiting disease, but it can progress to Guillain–Barré syndrome [3,4]. However, although the main mode of transmission is the bite of *Aedes aegypti*, since the outbreak in Brazil in 2015, sexual and congenital transmissions of ZIKV have been proven in 3720 cases, which can lead to congenital Zika syndrome [2,5,6,7,8]. Importantly, sexual transmission has been associated with the occurrence of ZIKV infection in nonendemic areas [9], with the persistence of ZIKV in female and male reproductive tracts [10,11]. Prolong shedding of RNA into the semen of symptomatic men has been demonstrated up to 9 months after the onset of symptoms; in contrast, infectious particles were seen only 30 days after the onset of the disease [12]. 

Corroborating this, a prospective case study of a Brazilian woman demonstrated the presence of the antigen and viral genome in the vagina and endocervical samples up to the 31st day of Zika onset symptoms [13]. The high permissiveness of reproductive tract cells to ZIKV in-vitro infection has also been demonstrated [14,15,16]; studies in different experimental models have also pointed to the relevance of vaginal mucosa cells for ZIKV infection [17,18,19,20]. These data have demonstrated that infected semen in vaginal and endocervical mucosa can contribute strongly to the sexual transmission cycle and complications caused by ZIKV infection, such as male infertility and, especially, those associated with pregnant women [5,21,22]. 

Despite the severe morbidity caused by Zika fever, there is still no specific treatment for the disease. Several approaches to drug repurposing have been conducted to accelerate anti-ZIKV drug discovery. In this context, antimalarial drugs have indicated a promising anti-ZIKV effect in in-vitro and in-vivo models [23,24,25,26]. These drugs are usually classified according to their source or chemical structure; among them, quinoline derivatives represent the most well-known antimalarial organic compounds, with antimicrobial, antifungal, analgesic, antitumor, and anti-inflammatory activities [27,28]. Some groups derived from quinoline are aminoquinolines, which include chloroquine, hydroxychloroquine, amodiaquine and primaquine, and amino alcohol groups (including mefloquine and quinine) [29]. 

Chloroquines have demonstrated to be potent inhibitors of ZIKV in-vitro and in-vivo infection models [24,25,26,30,31,32]. Other quinoline derivatives have also shown anti-ZIKV in-vitro effects, e.g., amodiaquine [33], hydroxychloroquine [34,35], mefloquine, quinacrine, and GSK369796 [23,36]. However, since several of these drugs can induce adverse reactions, the chemical structure of quinoline remains an important basis for the design of new compounds aimed at improving its effectiveness and reducing toxic effects [37,38,39,40]. 

Molecular hybridization is a medicinal chemistry tool often used to increase efficacy and minimize factors such as toxicity and drug resistance. The rational drug design allows the development of single hybrid molecules with dual functionality and/or targets, with the same or distinct mechanism of action from parent drugs. Moreover, a new generation of hybrids, instead of combined drugs, has the potential advantage of a lower risk of drug–drug adverse interactions and greater treatment adherence [41]. The antiplasmodial activity of hybrid compounds derived from chloroquine and sulfadoxine was evaluated in in-vitro and in-vivo experimental models [42]. The compounds showed high schizonticidal blood activity in vitro against a W2–chloroquine-resistant clone, with IC_50_ values ranging from 0.05 to 1.63 µM. Ten of them showed an IC_50_ (ranging from 0.05 to 0.40 µM) lower than the reference drugs chloroquine (IC_50_ = 0.46 µM) and sulfadoxine (IC_50_ > 15.5 µM), and four compounds exhibited higher SI values than chloroquine. Two compounds inhibited *P. berghei* parasitemia in vivo, by 47% and 49%, on day 5 after mice inoculation (20 mg/kg). The most active hybrid is considered a new prototype for the development of an antimalarial drug against chloroquine-resistant parasites [42].

Here, our aim is to investigate, for the first time, the anti-ZIKV effect of active antiplasmodial hybrid compounds derived from chloroquine and sulfadoxine. We demonstrate that all compounds are highly potent in inhibiting the ZIKV replication in human cervical cells in a dose-dependent manner and completely abolish the viral progeny at the maximum tested dose in cervical and Vero cell lines. However, further studies will be needed to investigate whether these new chemical structures can lead to the improvement of chloroquine antiviral activity. 

## 2. Materials and Methods 

### 2.1. Cell Cultures

Human cervical epithelial cell line (C33-A) and Vero cell line (from African green monkey kidney) was grown in Dulbecco’s modified Eagle medium (DMEM; Gibco, Waltham, MA, USA), supplemented with 10% fetal bovine serum (FBS; Sigma-Aldrich, St. Louis, MO, USA), 2% L-glutamine at 200 mM (Sigma-Aldrich, St. Louis, MO, USA), 1% sodium pyruvate at 100 mM (Gibco, Waltham, MA, USA), and 10,000 units/mL penicillin with 10 mg/mL streptomycin (Sigma-Aldrich, St. Louis, MO, USA), and maintained at 37 °C under a 5% CO_2_ atmosphere. Vero cells were also used to perform the plaque-forming reduction assays.

### 2.2. Virus

Brazilian Zika virus strain (GenBank access: KX212103) was propagated in a C6/36 cell line (from *Aedes albopictus* mosquito larvae), titrated by plaque assay (plaque-forming unit (PFU)/mL), and stocked in a freezer at −70 °C until the assays [43].

### 2.3. Compounds

The quinolinic derivatives *N*-(2-((7-chloroquinolin-4-yl)amino)alkyl)benzenesulfonamides (hybrid compounds derived from chloroquine and sulfadoxine, named C-Sd1 to C-Sd7; see Table 1) and mefloquine hydrochloride were synthesized at Institute Pharmaceuticals Technology/Farmanguinhos/FIOCRUZ, as described previously [42]. Briefly, the hybrid compounds were designed bearing the following pharmacophore moieties: 7-chloroquinoline, present in the chloroquine, and benzenesulfonamide, present in sulfadoxine; both drugs are used to treat malaria. The pharmacophore moieties were separated by a distinct linker group that is not found in the individual molecular frameworks of their parent drugs. The new hybrid compounds may contain three or four methylene carbon units (CH_2_) and substituents at the 4-position of the benzenesulfonamide moiety [42]. 

Mefloquine was used as control of C-Sds activity due to its potent anti-ZIKV activity, as demonstrated previously [23,36,39]. 

All compounds were solubilized in dimethyl sulfoxide (DMSO; Thermo Fisher Scientific, Waltham, MA, USA) to prepare stock solutions at 10 mM. The work solutions were prepared in culture medium immediately before the assays.

### 2.4. Cytotoxicity Assay 

Cell viability was assessed in mock-infected cervical and Vero cell cultures treated with C-Sds and the mefloquine drug (12 to 200 µM) or 2% DMSO (vehicle control) and maintained at 37 °C under a 5% CO_2_ atmosphere for 48 h. Thereafter, the cultures were incubated with 10% PrestoBlue reagent (Invitrogen, Carlsbad, CA, USA) for 4 h at 37 °C and the cell viability was measured at 570 and 600 nm, as recommended by the manufacturer, using a spectrophotometer VERSAmax (Molecular Device, San Diego, CA, USA). The percentage of viable cells was expressed, and CC_50_ (cytotoxic concentration that reduces 50% of cell viability) was calculated from a nonlinear regression analysis (dose–response curve) using GraphPad Prism 8.0 software (GraphPad Software Inc., San Diego, CA, USA).

### 2.5. Infection and Treatment

Cervical cell cultures were infected with the Brazilian ZIKV strain (ZIKV-BR) at multiplicities of infection (MOI) 1 and 10 by incubation for 2 h under gentle agitation every 15 min. Next, the cultures were washed 3 times to remove noninternalized virus and maintained at 37 °C under a 5% CO_2_ atmosphere for infection kinetic evaluation at 24, 48, and 72 h. 

To assess the antiviral effect, cervical cells were infected with ZIKV-BR at MOI 10 and Vero cells were infected with ZIKV-BR at MOI 1 by incubation for 2 h, with gentle agitation every 15 min. After this time, the cultures were washed 3 times to remove noninternalized virus and treated with nontoxic concentrations of C-Sds and mefloquine or with 0.12% or 0.25% DMSO (vehicle control) and maintained at 37 °C under a 5% CO_2_ atmosphere for 48 h. Next, the supernatants of both cultures were collected for a plaque-forming reduction assay, and the supernatants of cervical cell culture were collected for viral genome detection using an RT-qPCR assay. To observe ZIKV–envelope glycoprotein expression, the cervical cells were fixed in 4% paraformaldehyde (PFA; Sigma Aldrich, St. Louis, MO, USA) for analysis by immunofluorescence.

### 2.6. Antiviral Activity Assessments 

#### 2.6.1. Plaque-Forming Reduction Assay

The evaluation of plaque-forming reduction (PFR) was performed with supernatants obtained from infected–treated cervical and Vero cells. The supernatants were diluted (1:1000) to allow plaque quantification, and 100 µL/well was added to Vero cell cultures seeded in 24-well plates. After 1 h of incubation under gentle agitation every 15 min., the cultures were washed to remove noninternalized viruses and added to a semisolid medium containing 3% carboxymethyl cellulose (CMC; Sigma-Aldrich, St. Louis, MO, USA) for 7 days at 37 °C under a 5% CO_2_ atmosphere. Next, the cultures were fixed with 10% formaldehyde (Thermo Fisher Scientific, Waltham, MA, USA) and stained with 0.04% violet crystal (Sigma-Aldrich, St. Louis, MO, USA). For calculation purposes, we used (i) the percentage of plaque-forming reduction by the formula [1- (number of lysis plates in the treated/number of lysis plates in 0.25%DMSO)] × 100 [44]; (ii) the half-maximal effective concentration (EC_50_), which reduces 50% of plaque-forming, by nonlinear regression analysis (dose–response curve) using GraphPad Prism 8.0 software (GraphPad Software Inc., San Diego, CA, USA); (iii) a selectivity index (SI), a ratio that measures the window between cytotoxicity (CC_50_) and the antiviral effect (EC_50_).

#### 2.6.2. Viral Genome Detection

Inhibition of ZIKV replication in cervical cell cultures, infected and treated with 12 µM C-Sds and mefloquine, was assessed by directly measuring an RNA fragment by quantitative polymerase chain reaction (RT-qPCR). The reaction was performed using previously described ZK-F-E-Bonn (AGYCGYTGYCCAACACAAG) and ZK-R-E-Bonn (CACCARRCTCCCYTTGCCA) primers and a probe-target (6-FAM6-CCTMCCTYGAYAAG-CARTCAGACACYCAA-BHQ) [45]. The viral nucleic acids were extracted using the commercial diagnostic High Pure Viral Nucleic Acid kit (Roche Diagnostics Brazil Ltd.a., São Paulo, SP, BR). The cDNA and quantification were performed using the AgPath-ID One Step RT-qPCR kit (Thermo Fisher Scientific, Waltham, MA, USA) in a total volume of 20 µL. Each reaction consisted of 12.5 µL of 1× buffer (PCR Buffer), 2.5 µL of sense primers (1 µM), 2.5 µL of antisense primers (1 µM), 2 µL (0.4 µM) of the specific probe, and 1 µL of 1x enzyme. A synthetic standard curve (TTCGTCACCARRCTCCCYTTGCCACGTATTTGRGTGTCTGAYTGCTTRTCRAGGKAGGATGCGTCTTGTGTTGGRCARCGRCTCTGATA) was designed for absolute viral load quantification, with limited detection of 10^2^ copies/mL, R2 = 0.989, and slope = 3.453.

#### 2.6.3. Viral Protein Expression Detection

ZIKV–envelope glycoprotein was detected by immunofluorescence in cervical cell cultures infected and treated with 12 µM C-Sds and mefloquine. Fixed cultures with 4% PFA were incubated with 3% bovine serum albumin (Thermo Fisher Scientific, Waltham, MA, USA) for 30 min. At room temperature, the cultures were washed 3 times and incubated with monoclonal antibody-specific 4G2 anti-flavivirus (LATAM/Biomanguinhos) for 1 h at 37 °C. Thereafter, the cells were washed 3 times and incubated with AlexaFluor 488-conjugated secondary antibody for 1 h at 37 °C (Thermo Fisher Scientific, Waltham, MA, USA) to reveal the expression of viral glycoprotein by green staining. In addition, 4′,6-diamidino-2-phenylindole dihydrochloride (DAPI; Sigma-Aldrich, St. Louis, MO, USA) was used to stain the nuclei of the host cells in blue, and then the samples were mounted with antifading 2.5% 1,4-diazabicyclo[2.2.2]octane (DABCO; Sigma-Aldrich, St. Louis, MO, USA). The samples were observed under an Axio Observer Z1 motorized inverted fluorescence microscope (Carl Zeiss, Oberkochen, Germany). The images were shown in DIC (differential interference contrast) microscopy. The cell’s quantification was performed by CellProfiler software (CellProfilerTM cell image analysis) to calculate (i) the percentage of infected cells using the formula 100 × (cells expressing viral protein/accounted cells total) and (ii) the percentage of infection reduction using the formula [1 − (percentage of infected cells and C-Sds treated/percentage of infected cells in 0.12% DMSO)] × 100. 

### 2.7. Statistical Analysis

All statistical tests were performed using a one-way analysis of variance (ANOVA) with Dunnett’s multiple comparisons test from GraphPad Prism 8.0 software (GraphPad Software Inc., San Diego, CA, USA), where *p* < 0.007 and *p* < 0.0001 represent the levels of significance. The data are representative of 3 to 5 independent assays run in duplicate.

## 3. Results

### 3.1. Cytotoxic Effect of C-Sds in Cervical and Vero Cell Lines

**Figure 1:**
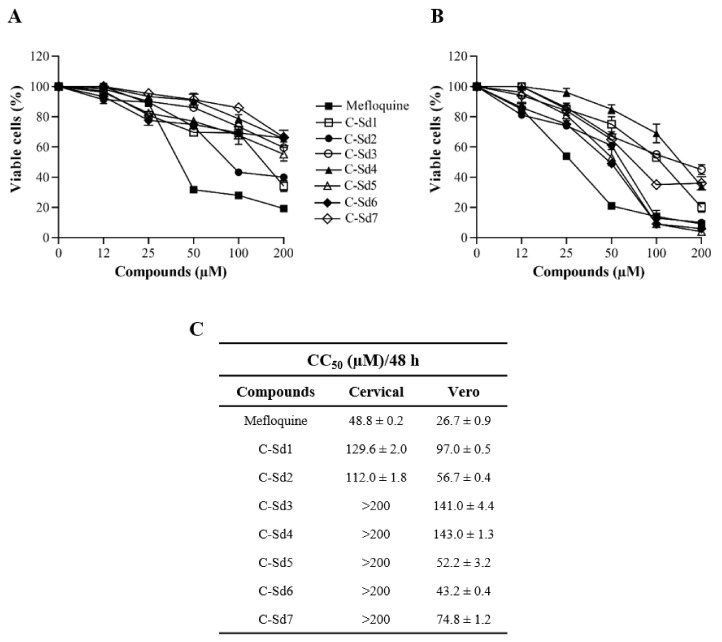
Kinetics of cellular viability of C-Sds hybrids and mefloquine were evaluated by PrestoBlue assay. The cervical and Vero cell lines were treated with C-Sds and mefloquine at different concentrations for 48 h. The graphs represent the mean ± standard deviation of the percentage of viable (**A**) cervical cells and (**B**) Vero cells. (**C**) The table shows the values of CC_50_/48 h of C-Sds and mefloquine. The data are representative of 3–5 experiments run in duplicate.

To define the concentrations to be used in the antiviral activity evaluation, the cytotoxic effect of C-Sds on cells was first determined by PrestoBlue reagent. The reduction of resazurin reagent by metabolically viable active cells was measured using a spectrophotometer. The concentration curve of the drug vehicle (0.5, 1 and 2% DMSO) was performed to test its self-effect. The treatment of mock-infected cervical and Vero cell cultures with 2% DMSO showed the viability of 89.1% ± 1.5% and 74.6% ± 1.0% of cells, while with 0.5%, it was 100.0% ± 1.7% and 100.0% ± 1.3% of viability, respectively (data not shown). The kinetics of cell viability showed a gradual reduction correlated to dose in all C-Sds and mefloquine in cervical cells (Figure 1A) and Vero cells (Figure 1B). It is possible that the decrease of cellular viability, observed in Figure 1 for both cell types, is, at least in part, due to the toxic effect of 2% DMSO at 200 µM. However, the C-Sds antiviral activity was performed at 25 µM, which corresponds to 0.25% DMSO. Since all compounds maintained the cell viability above 80% at 12 µM for both cell lines, we believe that it was not necessary to test cytotoxicity at concentrations lower than 6 µM. In this way, the range of nontoxic concentrations for subsequent assays was established as 6 to 25 µM for C-Sds and 6 to 12 µM for mefloquine to ensure the safety of the treatment. Regarding the CC_50_/48 h values for cervical cells, they were >200 µM for most of the C-Sds, except for C-Sd1 and C-Sd2, which showed CC_50_ values of 129.6 ± 2.0 and 112.0 ± 1.8 µM, respectively (Figure 1C). The CC_50_/48 h values for Vero cells ranged between 43.2 ± 0.4 to 143.0 ± 1.3 µM for all compounds (Figure 1C), while the CC_50_/48 h values of mefloquine for cervical cells was 48.8 ± 0.2 and 26.7 ± 0.9 µM for Vero cells (Figure 1C). These results showed that all C-Sds exhibited a lower cytotoxic effect than mefloquine since the CC_50_ values of most hybrid compounds was about 4-fold higher compared to the CC_50_ values of mefloquine, used as the control of C-Sds antiviral activity. 

### 3.2. ZIKV Infection Kinetics in Cervical Cell Line 

**Figure 2:**
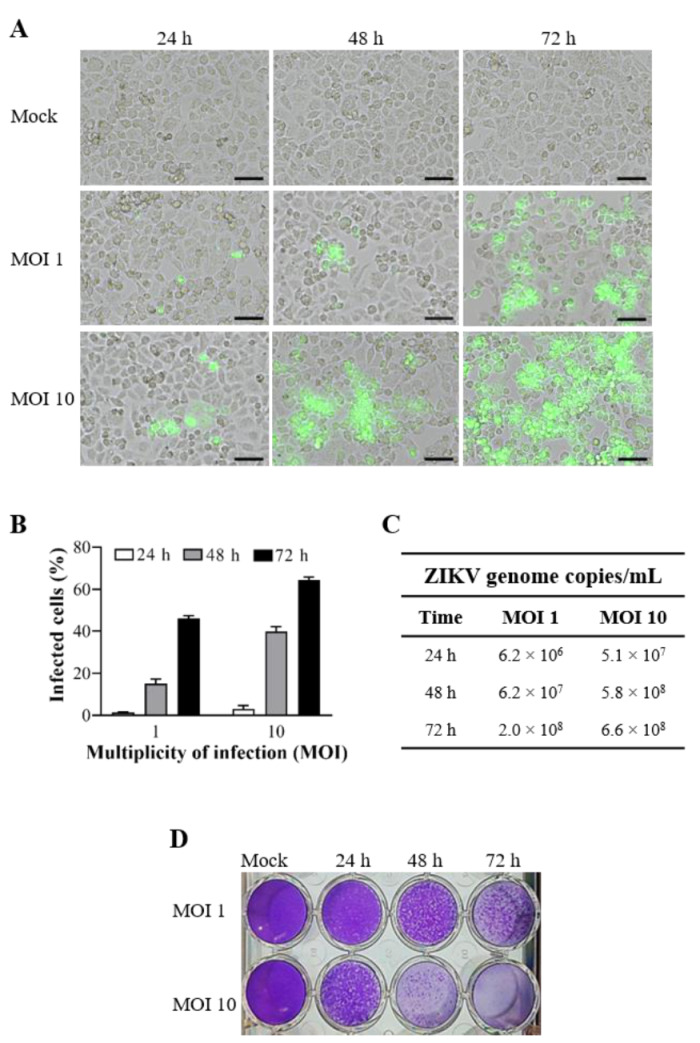
Kinetics of the Brazilian ZIKV strain (ZIKV-BR) infection in human cervical cell line. The cells were mock-infected or ZIKV-infected at MOI 1 and 10 for 24, 48, and 72 h. (**A**) ZIKV–envelope glycoprotein expression (green) was detected by immunofluorescence and images were capture in differential interference contrast microscopy. (**B**) The graph represents the mean ± standard deviation of the percentage of infected cells during the kinetics of infection. The supernatants of cell cultures were collected to evaluate (**C**) viral load by RT-qPCR and (**D**) plaque-forming by plaque assay. The data are representative of 3–5 experiments run in duplicate. Bars, 50 µm.

To determine the infection rate of ZIKV-BR in cervical cells, we performed the infection kinetics at different MOIs and times. The cultures were infected with ZIKV-BR at MOIs 1 and 10 for 24, 48, and 72 h (Figure 2). We observed that cervical cells were permissive to ZIKV infection, as demonstrated by intracellular staining of the ZIKV–envelope glycoprotein (Figure 2A) and the percentage of infected cells (Figure 2B), indicating an increase of viral replication according to MOI and time postinfection. The results revealed the percentage of infected cells at MOI 10 of 3.1% ± 1.4%, 39.9% ± 2.3%, and 64.4% ± 1.5% after 24, 48, and 72 h of infection, respectively (Figure 2B). The viral load determined in the supernatant of infected cultures with MOI 1 reached 2.0 × 10^8^ copies/mL only 72 h postinfection, while with MOI 10, the value was already 5.8 × 10^8^ copies/mL at 48 h postinfection (Figure 2C). Kinetics of infection MOI and time-dependence were also observed by plaque-forming assay (Figure 2D). Thus, the anti-ZKV activity of hybrid compounds was determined with the infected cultures at MOI 10 for 48 h, which already maintains high levels of infection. 

### 3.3. C-Sds Inhibit ZIKV Growth in Cervical and Vero Cell Lines 

**Figure 3:**
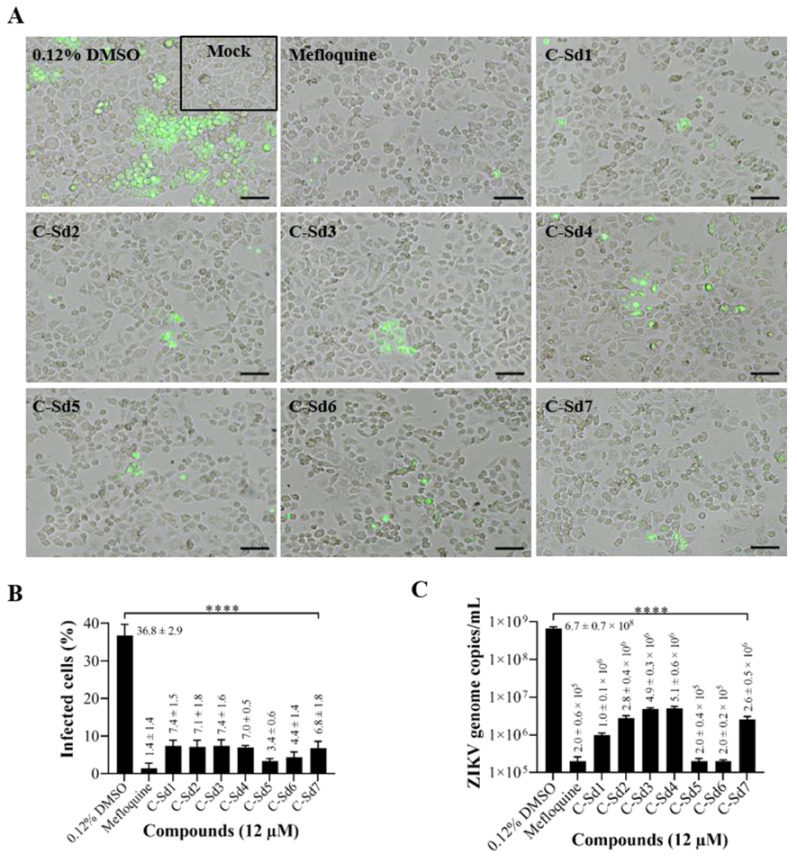
Evaluation of the C-Sds antiviral effect by immunofluorescence and RT-qPCR. The cervical cells were mock-infected or ZIKV-infected at MOI 10 and treated with 12 µM of C-Sds or mefloquine for 48 h. The images are shown in DIC (differential interference contrast) microscopy; (**A**) fluorescent intracellular staining shows the ZIKV–envelope glycoprotein (green). The graphs represent the mean ± standard deviation of (**B**) the percentage of infected cells and (**C**) copies of the ZIKV genome/mL. The data are representative of 3–5 experiments run in duplicate. The statistical significance was determined by one-way ANOVA, followed by Dunnett’s multiple-comparisons test. **** *p* < 0.0001 for all C-Sds compared to control. Bars, 50 µm.

**Figure 4:**
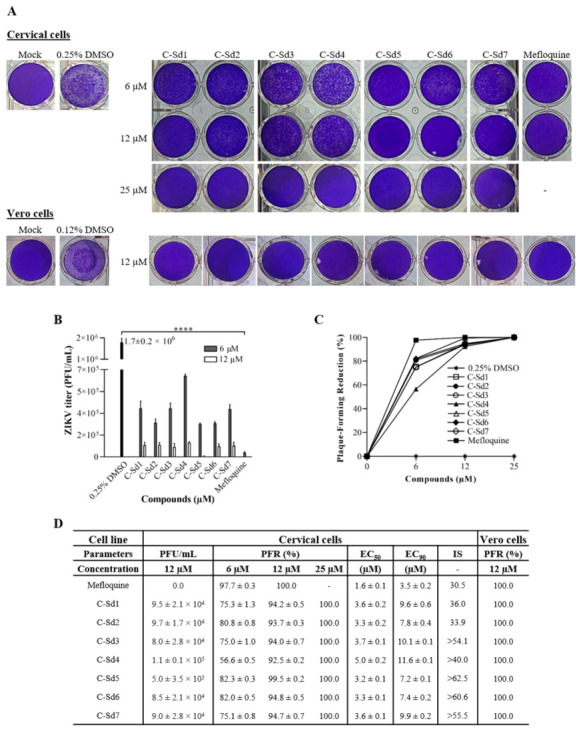
Evaluation of anti-ZIKV effect in cervical and Vero cell lines by plaque assay. The cervical cells were mock-infected or ZIKV-infected at MOI 10 and treated with 6, 12, and 25 μM of C-Sds or 6 and 12 μM of mefloquine for 48 h. The Vero cells were mock-infected or ZIKV-infected at MOI 1 and treated with 12 μM of C-Sds or mefloquine for 48 h. (**A**) The cultures’ supernatant was collected and diluted (1:1000) for the plaque assay. The graphs represent the mean ± standard deviation of (**B**) the ZIKV titer (PFU/mL) and (**C**) the dose-response curve of PFR%. (**D**) The table summarizes the parameters of antiviral activity evaluation in cervical and Vero cells. The data are representative of 3–5 experiments run in duplicate. The statistical significance was determined by one-way ANOVA, followed by Dunnett’s multiple-comparisons test. **** *p* < 0.0001.

Infection parameters were measured to assess the antiviral activity of C-Sds and mefloquine in cervical and Vero cells infected with ZIKV-BR (Figure 3 and Figure 4). Immunofluorescence analysis of ZIKV–envelope glycoprotein expression in cervical cells infected and treated with all compounds at 12 µM demonstrated a strong reduction of infected cells when compared to intracellular staining in the control (0.12% DMSO; see Figure 3A). The treatment induced a reduction of 79.8% ± 4.2% to 90.7% ± 1.5% of infected cells as compared to 36.8% ± 2.9% of infection in control cultures (0.12% DMSO). The treatment with C-Sd5 reduced 90.7% ± 1.5% of infected cells, comparable to 96.3% ± 3.7% of reduction by mefloquine treatment (Figure 3B). The inhibitory effect of C-Sds was also demonstrated by measuring viral loads in the supernatants of cervical cell cultures treated at 12 µM (Figure 3C). A high viral load of 6.7 ± 0.7 × 10^8^ viral genome copies/mL detected in the supernatant of control cultures allowed the evaluation of the stringent effect of the compounds. The data of released viral RNA, obtained in cultures treated with C-Sd1, C-Sd2, C-Sd3, C-Sd4, and C-Sd7, showed 2 logs reduction of the viral load when compared to the control, exhibiting values of 1.0 ± 0.1 × 10^6^, 2.8 ± 0.4 × 10^6^, 4.9 ± 0.3 × 10^6^, 5.1 ± 0.6 × 10^6^, and 2.6 ± 0.5 × 10^6^ copies/mL, respectively (Figure 3C). Importantly, the treatment with C-Sd5 and C-Sd6 at 12 µM reduced 3 logs of viral load, exhibiting values of 2.0 ± 0.4 × 10^5^ and 2.0 ± 0.2 × 10^5^, respectively, comparable to 2.0 ± 0.6 × 10^5^ copies/mL with the mefloquine treatment (Figure 3C).

The quantification of plaque-forming units (PFU/mL) in supernatants obtained from cervical cells treated with 6 to 25 µM of C-Sds and 6 and 12 µM of mefloquine was performed to calculate plaque-forming reduction percentage (PFR%), EC_50_, and SI values (Figure 4). The viral title of 1.7 ± 0.2 × 10^6^ PFU/mL in the control cultures (0.25% DMSO) was reduced by C-Sds and mefloquine treatment in a dose-dependent manner (Figure 4A,B). The potent activity in inhibiting ZIKV replication of all compounds revealed over 50% and 90% of PFR at 6 and 12 µM, respectively, while 25 µM of C-Sds and 12 μM of mefloquine inhibited 100% of viral progeny (Figure 4C,D). The % of PFR analysis showed higher antiviral activity of mefloquine at 6 μM than C-Sds (*p* < 0.007). The antiviral activity was also evaluated in Vero cells treated with all compounds at 12 and 25 µM. Here, the kinetics infection of Vero cells was not performed, as we previously described elsewhere [46]. Infection of Vero cells with ZIKV-BR at MOI 1 reached 30.3% ± 2.5% of infected cells within 48 h (data not shown), comparable to 36.8% ± 2.9% of infected cervical cells at MOI 10 (Figure 3B,D). The plaque-forming reduction analysis showed that 12 μM of C-Sds and mefloquine in Vero cells led to 100% of PFR (Figure 4A,D), confirming the high anti-ZIKV efficacy of all compounds in both cell types.

Regarding effective concentration in cervical cells, EC_50_ was similar among all C-Sds, reaching values around 3 μM, except for C-Sd4 (5.0 ± 0.2 μM), while EC_90_ ranged from 7.2 ± 0.1 to 11.6 ± 0.1 µM (Figure 4D). For mefloquine, the EC_50_ and EC_90_ values were 1.6 ± 0.1 and 3.5 ± 0.2 µM, respectively, showing higher antiviral activity than C-Sds (Figure 4D). The selectivity index was over 40 for most C-Sds; however, SI for C-Sd5 was >62.5, and for C-Sd6, it was > 60.6, indicating an excellent therapeutic window compared to SI 30.5 for mefloquine (Figure 4D). The results suggest that the reference drug exhibits a cytotoxic effect superior to C-Sd5 and C-Sd6; however, an additional approach will be needed to confirm the data.

## 4. Discussion

Currently, drugs used to treat malaria have shown effects against emerging viruses, such as the Zika virus [25,32], the dengue virus [47,48,49], and the Chikungunya virus [50,51]. Here, we analyze the anti-ZIKV effect of seven hybrid compounds derived from chloroquine and sulfadoxine antimalarial drugs, and, for the first time, we showed the high effectiveness of these quinolinic derivatives against ZIKV-BR in cervical and Vero cells, with 100% viral progeny inhibition. 

The hybrid compounds tested herein against ZIKV were designed to improve antimalarial drugs and exhibited a stronger antiplasmodium activity when compared to chloroquine [42]. Indeed, when tested in vitro against *P. falciparum*, most compounds showed an EC_50_ value (ranging from 0.05 to 0.40 µM) lower than the reference drugs chloroquine (IC_50_ = 0.46 µM) and sulfadoxine (IC_50_ > 15.5 µM), and some compounds exhibited higher SI values than chloroquine, including the compound we named C-Sd5, which was also the most potent in inhibiting *P. berghei* parasitemia in infected mice [42]. In this study, the most active hybrid is considered a new prototype for the development of an antimalarial drug against chloroquine-resistant parasites. 

Our results demonstrate that the cytotoxicity effect of C-Sds in cervical and Vero cell lines occurred in a dose-dependent manner, with CC_50_ values ranging from 43.2 ± 0.4 to >200 µM, being more toxic in the Vero cell line. All compounds were safe at a dose below 12 μM. The cytotoxicity effect of hybrid compounds was first assessed by neutral red assay (NR) in buffalo green monkey (BGM) cells and showed only a slight difference of CC_50_ values, ranging from 14.9 ± 0.0 to 338.6 ± 48.3 μM [42]. Mefloquine cytotoxicity was about 4-folder higher than C-Sds by PrestoBlue reagent, with Vero cells again being more susceptible (26.7 ± 0.9 µM). However, the cytotoxic effect of mefloquine in Vero cells is controversial; while one study showed a CC_50_ value of 20.4 ± 2.2 µM [23], similar to that observed in our results, another showed CC_50_ of 212 ± 14 µM [39]. In addition, in baby hamster kidney cells (BHK-21), the CC_50_ value of 17.72 ± 3.5 µM was even lower than that found by us [23].

ZIKV-BR was infective for the cervical cell line, as detected by plaque-forming, viral glycoprotein expression and released RNA in a dose- and time-dependent manner. Cervical cells were more resistant to ZIKV-BR than Vero cells, which, to maintain similar infection levels, were infected at MOI 1. In fact, different susceptibility profiles of human and animal cell lines have been described [52]. ZIKV replicates efficiently in Vero cells, which are widely used for virus production and isolation, whereas in some human cell lines, ZIKV replicates well but can induce strong inflammatory cytokine and chemokine production and interfere in infection [46,53].

The antiviral activity of C-Sds demonstrated a strong reduction of infected cells and released RNA when compared to control (0.12% DMSO). Importantly, a high viral load of 6.7 ± 0.7×10^8^ copies/mL detected in the supernatant of control cultures allowed the evaluation of the stringent effect of the compounds. Literature data support the importance of evaluating antiviral activity upon high viral load, which will be correlated with EC_50_ and SI values. Thus, viral load has been considered an important factor in defining therapeutic management, such as the need to initiate preventive therapy, treatment duration, and prognostication [54,55]. 

The high anti-ZIKV efficacy of compounds was confirmed through the dose–response curve of plaque-forming reduction. All hybrid compounds inhibited over 90% of infectious viral release at 12 μM in both cell types (Figure 4). While our results obtained from Vero cells infected with ZIKV-BR at MOI 1 revealed that all hybrid compounds inhibited 100% of viral progeny at 12 μM (Figure 4), Barbosa-Lima et al. found only 50% of viral inhibition at 12 ± 3.2 μM of chloroquine in Vero cells infected with ZIKV-BR at MOI 0.1 [31]. 

As hoped, due to a more parasite-oriented action, in our study, EC_50_ of C-Sds anti-ZIKV were higher than EC_50_ for anti-plasmodium activity (0.05 to 1.63 µM) [42]. The treatment of infected cervical cells showing EC_50_ values ranging from 3.2 ± 0.1 to 5 ± 0.2 μM for all C-Sds and 1.6 ± 0.1 μM for mefloquine. The SI obtained by treatment with hybrid compounds ranged from 33.9 to >62.5, and, for mefloquine, it was 30.5 (Figure 3). Mefloquine antiviral activity has also been shown to reduce infection of ZIKV strain MR766 in Vero cells with values of EC_50_ 3.9 ± 0.2 µM, CC_50_ 20.4 ± 2.2 µM, and SI 5.17 µM [23]. Moreover, the anti-ZIKV activity of chloroquine has been tested in different cell types, infected with different ZIKV strains (MR766, GZ02, PLCal ZV, PRVABC59, and ZIKV BR), and multiplicities of infection (MOIs 0.1, 1, and 2) [25,26,30,31,32]. In these studies, EC_50_ values ranged from 5.31 ± 0.64 to 14.20 ± 0.18 μM, and the highest SI value was 34. Importantly, the higher the SI value, the more effective and safer a drug must be during in-vivo treatment. In general, the SI values of C-Sds were higher than mefloquine and those reported in the literature for chloroquine [25,26,30,31,32]. However, further studies will be needed to investigate whether these new chemical structures can lead to the improvement of chloroquine and mefloquine antiviral activity. 

C-Sd5 was one of the most active compounds previously tested against plasmodium, exhibiting EC_50_ and SI values that were better than chloroquine, and it was considered a new prototype for the development of an antimalarial drug against chloroquine-resistant parasites [42]. Interestingly, C-Sd5 and C-Sd6 were also most active against ZIKV, reducing infected cells (over 88%) and virus RNA release (3-logs) at 12 µM and infectious virus release (about 82%) and SI values (>62.5 and >60.6, respectively) at 6 µM. Importantly, the reduction of infected cells and viral load induced by C-Sd5 was comparable to mefloquine at 12 µM. 

It is possible to observe some common chemical structure characteristics in C-Sd5 and C-Sd6; in both compounds, the 7-chloroquinoline and benzenesulfonamide moieties are linked by four CH_2_ units and have a butyl group in common, such as the C-Sd7, which also showed good antiviral activity. The quinolinic derivatives C-Sd5, C-Sd6, and C-Sd7 (Table 1) contain halogens in their structures that seem to increase antiviral activity since this is the only difference between them and C-Sd4, which demonstrated the least effect of the inhibition of infectious viral particles at 6 µM. In fact, the improvement of these compounds may be associated with the presence of halogens that confer a more lipophilic character or greater chemical reactivity, depending on their position and nature [56]. On the other hand, independent of halogen’s presence in the chemical structure, the linkage of the 7-chloroquinoline and benzenesulfonamide moieties by three CH_2_ units and the presence of a propyl group in the quinolines C-Sd2 and C-Sd3 may have conferred a greater cytotoxic effect for those derived, which had the lowest SI values.

In conclusion, the data showed that the new hybrid compounds were highly potent against in-vitro ZIKV infection. These compounds look promising for future studies related to the improvement of chloroquine activity and the development of antiviral prototypes for Zika fever treatment.

## Figures and Tables

**Table 1:** Chemical characteristics of hybrid compounds derived from the chloroquine and sulfadoxine drugs.(C-Sds) and mefloquine hydrochloride.

C-Sds	Formula	Nomenclature	Structure	Molecular Weight
**C-Sd1**	C_19_H_20_ClN_3_O_2_S	*N*-(3-((7-chloroquinolin-4-yl)amino)propyl)-4-methyl-benzenesulfonamide	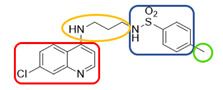	389.0965
**C-Sd2**	C_18_H_17_BrClN_3_O_2_S	4-Bromo-*N*-(3-((7-chloroquinolin-4-yl)amino)propyl)-benzenesulfonamide	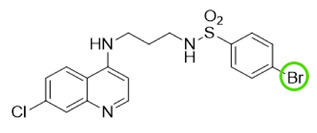	452.9913
**C-Sd3**	C_18_H_17_ClFN_3_O_2_S	*N*-(3-((7-chloroquinolin-4-yl)amino)propyl)-4-fluorobenzenesulfonamide	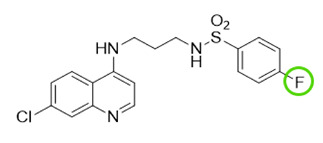	393.0714
**C-Sd4**	C_19_H_20_ClN_3_O_2_S	*N*-(4-((7-chloroquinolin-4-yl)amino)butyl)-benzenesulfonamide	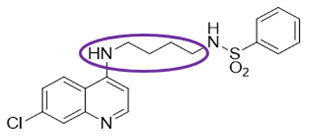	389.0965
**C-Sd5**	C_19_H_19_Cl_2_N_3_O_2_S	4-Chloro-*N*-(4-((7-chloroquinolin-4-yl)amino)butyl)-benzenesulfonamide	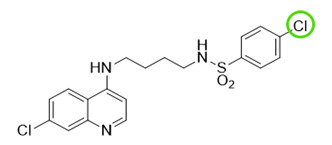	423.0575
**C-Sd6**	C_19_H_19_BrClN_3_O_2_S	4-Bromo-*N*-(4-((7-chloroquinolin-4-yl)amino)butyl)-benzenesulfonamide	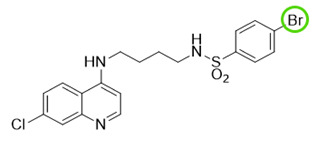	467.0070
**C-Sd7**	C_19_H_19_ClFN_3_O_2_S	*N*-(4-((7-chloroquinolin-4-yl)amino)butyl)-4-fluorobenzenesulfonamide	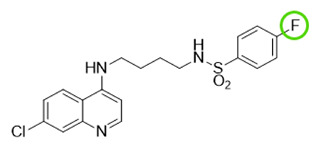	407.0871
**Mefloquine hydrochloride** C_17_H_17_ClF_6_N_2_O		(R)-(2.8-bis(trifluoromethyl)quinolin-4-yl)((S)-piperidin-2-yl)methanol hydrochloride	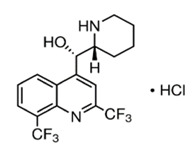	414.0934

7-chloroquinoline and benzenesulfonamide moiety. ligant with three methylene carbon units (CH_2_). ligant with four CH_2_ units and substituents methyl and halogens.

## Data Availability

No new data were created or analyzed in this study. Data sharing is not applicable to this article.
